# Fast Determination of Flip Angle and T_1_ in Hyperpolarized Gas MRI During a Single Breath-Hold

**DOI:** 10.1038/srep25854

**Published:** 2016-05-12

**Authors:** Jianping Zhong, Weiwei Ruan, Yeqing Han, Xianping Sun, Chaohui Ye, Xin Zhou

**Affiliations:** 1 Key Laboratory of Magnetic Resonance in Biological Systems, State Key Laboratory for Magnetic Resonance and Atomic and Molecular Physics, National Center for Magnetic Resonance in Wuhan, Wuhan Institute of Physics and Mathematics, Chinese Academy of Sciences, Wuhan 430071, China; 2 School of Physics, Huazhong University of Science and Technology, Wuhan 430074, China

## Abstract

MRI of hyperpolarized media, such as ^129^Xe and ^3^He, shows great potential for clinical applications. The optimal use of the available spin polarization requires accurate flip angle calibrations and *T*_*1*_ measurements. Traditional flip angle calibration methods are time-consuming and suffer from polarization losses during *T*_*1*_ relaxation. In this paper, we propose a method to simultaneously calibrate flip angles and measure *T*_*1*_ *in vivo* during a breath-hold time of less than 4 seconds. We demonstrate the accuracy, robustness and repeatability of this method and contrast it with traditional methods. By measuring the *T*_*1*_ of hyperpolarized gas, the oxygen pressure *in vivo* can be calibrated during the same breath hold. The results of the calibration have been applied in variable flip angle (VFA) scheme to obtain a stable steady-state transverse magnetization. Coupled with this method, the ultra-short TE (UTE) and constant VFA (CVFA) schemes are expected to give rise to new applications of hyperpolarized media.

Hyperpolarized gas MRI has become a useful tool for lung gas space imaging^1^. Compared to the Boltzmann equilibrium state, hyperpolarized methods can enhance nuclear magnetization by a factor of 10^4^ to 10^5^ 2. Thus relatively high signal-to-noise ratio (SNR) images can be acquired even by inhaling a small dose of gas (^3^He, ^129^Xe, or ^83^Kr)^3^. The gas distribution in lungs during breath hold enables mapping of visual ventilation defects in diseased lungs such as COPD^4^ and asthma^5^. By measuring the spin-lattice relaxation time (*T*_*1*_) of the gas, the partial oxygen pressure can be determined^6^,^7^,^8^, which is an important parameter related to the efficiency of gas exchange in the lung. These pre-clinical studies illustrate the potential of hyperpolarized gas MRI for clinical applications.

The nonrenewable nuclear magnetization of hyperpolarized substances makes the SNR and resolution of lung images sensitive to the choice of flip angle^9^. Some studies^10^ sought to maximize SNR by choosing an optimal constant flip angle, while others^11^,^12^ focused on designing a VFA scheme. Both approaches result in a trade-off between SNR and image quality. In either case, the accurate calibration of flip angles is important for any optimal protocols of hyperpolarized MRI.

Traditional flip angle calibration methods in ^1^H NMR experiments, such as the “Spin Echo-Stimulated Echo (SE-STE)” method^13^ implemented on commercial NMR systems, are not suitable for hyperpolarized MRI, due to the rapid and non-optimal consumption of polarization by numerous big-angle RF pulses. Special calibration procedures adapted for hyperpolarized MRI have been proposed based on phase methods^14^,^15^,^16^,^17^ such as the Bloch–Siegert shift^14^,^15^ and Spatial Modulation of Magnetization (SPAMM)^16^. The Bloch–Siegert shift method calculates flip angles via the accumulated phase shift during an off-resonance RF pulse, showing good robustness in different situations. However, it is limited by the long Fermi pulse between on-resonance excitation and signal acquisition. This long pulse increases specific absorption rate and leads to decay of the transverse signal. The SPAMM method is not sensitive to flip angles smaller than 45° and has limited SNR due to the need to acquire two images separated by a so-called “SPAMM preparation” period done within a single breath-hold.

The standard method to calibrate pulses is based on a series of RF excitations of variable amplitudes or durations followed by fitting the resulting sine curve to the signal magnitudes^18^,^19^,^20^. However, these magnitude-based methods are susceptible to relaxation effects. Moreover, the measurement of *T*_*1*_ requires a pulse flip angle calibration. Most previous studies neglected or avoided the effects of *T*_*1*_ relaxation while calibrating flip angles^18^,^19^,^21^,^22^ and regarded flip angles as known values during *T*_*1*_ measurement^20^,^23^,^24^,^25^,^26^. However, these studies generally require more than two breaths: one for the flip angle calibration, and another one for the *T*_*1*_ measurement. In previous works^27^,^28^, special coils are used to evaluate excitation voltages of ^3^He (or ^129^Xe) by multiplication of the voltages from a ^1^H (or ^23^Na) coil using a suitable factor. In this case, the extra breath-hold for the flip angle calibration is not required. However, this method requires a specific coil. Thus it is not suitable for conventional cases. Another study^29^ calculates flip angles via thermally polarized samples, but the different load of the coil during *in vivo* study will affect the excitation voltages, which may generate substantial errors. Max *et al.* provided a method^30^ for the simultaneous estimation of *T*_*1*_ and the flip angle in a single scan through acquisition at non-regular time intervals. However, the result of *T*_*1*_ estimation was significantly affected by the assumed flip angle, and the long acquisition time needed for mounting time intervals also limits its usefulness during *in vivo* applications.

In this work, we propose a novel method to simultaneously calibrate flip angles and measure *T*_*1*_ of hyperpolarized gas during a short single breath-hold *in vivo*. The so-called single-breath method is magnitude-based and simple to use. It can be readily implemented on any standard MRI scanner without any hardware modification. The time needed for calibration is less than 4 s, which is much shorter than common breath-hold time (~16 s) for humans. We compared this method with conventional methods, and demonstrated its robustness and repeatability.

## Methods

### Theory

In the constant flip angle (CFA) sequence, the transverse signal (S_k_) of hyperpolarized media decays with each RF excitation according to


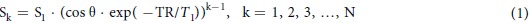


where k is the excitation number, N is the total number of excitations, S_1_ is the 1^st^ transverse signal, θ is the constant flip angle, TR is the repetition time and *T*_*1*_ is the longitudinal relaxation time. In hyperpolarized gas MRI, *T*_*1*_ refers to *T*_*1*_[pO_2_] to denote its dependence on the regional oxygen partial pressure, pO_2_. This interaction normally takes the form^31^


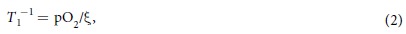


where ξ is called the oxygen enhancement factor (OEF), which depends on the magnetic field and temperature. The whole lung oxygen pressure can be calibrated by Eq. (2) provided that *T*_*1*_ is known.

In the MRI scanner the flip angles can be controlled by transmitter voltages or transmitter gain (TG). The TG setting (in dB units) for each angle is given by


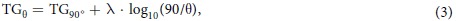


where TG_90°_ is the transmitter gain of a 90° pulse, λ is a factor close to 20. When TG decreased by 6.02 dB, the transmitter voltage was increased twice, but the flip angle was not exactly increased twofold because of B_1_ field inhomogeneity and transmission losses between RF coil and transmitter electronics^10^, which are considered as nonlinearities in Bloch equations. Moreover, for different shape of RF pulse, the factor λ in Eq. (3) is not exactly equal to 20. Thus the flip angle calibration contains not only the accurately measured TG_90°_ but also the measurement of λ.

**Figure 1:**
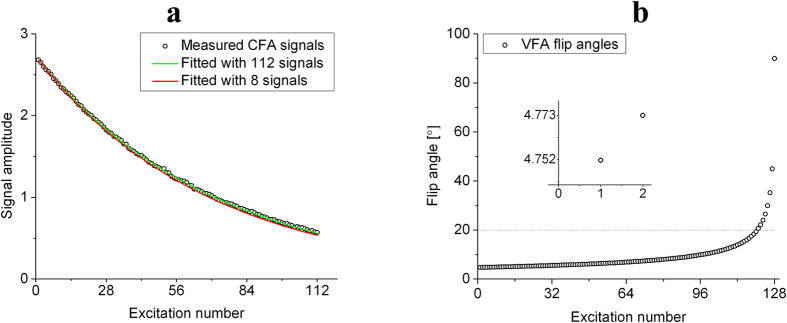
Theory of calibrating flip angles by magnitude-based methods. **(a)** Measured signals by constant flip angle scheme and the fitted lines by Eq. (1) with different N values. When N = 8 (the red line), the fitted value of the constant flip angle is 8.592°, which is very close to the actual value (8.475°) when N = 112 (the green line). Thus a large N value is not necessary in flip angle calibration. **(b)** Simulated flip angles of 128 excitations in the VFA scheme. Most of angles are smaller than 20°, and the difference between the 1^st^ and 2^nd^ angle is very small (0.021°). Thus accurate calibration of the angles smaller than 20° is important in the VFA scheme.

When calibrating flip angles by magnitude-based methods, any unknown flip angle can be fitted by the measurement of transverse signals according to Eq. (1), as shown in Fig. 1a. The fitted value can be considered to be the true value provided that N is large. In fact, the fitted flip angle value (8.592°) when N = 8 is very close to the actual value (8.475°) when N = 112. Thus we can obtain m angles just with 8 × m excitations, by using m different transmitter gains.

However, the m angles cannot be too large due to the nonreversible loss of polarization from RF pulses. In this work, the values of m angles were chosen between 3° to 20° according to the importance of small angles in the variable flip angle (VFA) scheme. The VFA scheme is important in 2D-FLASH, steady-state free precession (SSFP)^32^ and UTE methods because it can utilize polarization sufficiently and to generate steady transverse signals. The scheme was implemented by gradually increasing the flip angles of the form


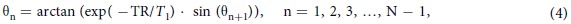


where n is the excitation number, N is the total number of excitations, and θ_N_ = 90°. As shown in Fig. 1b, the difference between the 1^st^ angle and 2^nd^ angle in VFA scheme is only 0.021°, and most angles are smaller than 20°. Thus it is very important to verify the accuracy of controlling small flip angles, especially those smaller than 20°. The purpose of calibration in this work is to measure the unknown parameters TG_90°_ and λ in Eq. (3) using m different RF pulses chosen between 3° and 20°. There are 8 excitations for each angle.

### Sequence design

**Figure 2:**
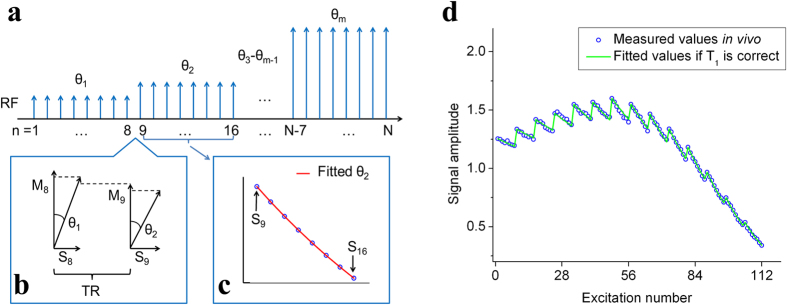
The sequence designed for fast estimation of flip angle and *T*_1_ during a single-breath. **(a)** The time sequence of single-breath calibration method. Calibration begins with a flip angle θ_1_ in the range 3°–6°, gradually increased to θ_m_ around 20°, with 8 excitations for each angle. The lengths of pulses were kept identical, and different angles were controlled by variable transmitter gains. **(b,c)** The two methods for fitting the unknown flip angles after setting an initial *T*_*1*_ value. Once *T*_*1*_ is correct, the fitted θ_2_ by method (**b**) should be equal to that by method (**c**). **(d)** Measured transverse signals during the single-breath calibration *in vivo* and the fitted values if *T*_*1*_ is correct. The mean measurement error of transverse signals was ±0.7%.

Our proposed method for calibration in a single-breath is shown in Fig. 2a. First, we set the TG_1_ value such that we get a small flip angle θ_1_ in the range 3°–6°. After 8 excitations by θ_1_, the transmitter gain is changed to a smaller value TG_2_ to obtain a bigger flip angle θ_2_. Subsequently, we set TG_3_ ~ TG_m_ to obtain gradually larger flip angles θ_3_ ~ θ_m_, 8 excitations for each angle. Finally, the flip angle θ_m_ is set to a value around 20° due to two reasons: 1) the signals S_N−7_ ~ S_N_ may decay to zero if θ_m_ became bigger than 20°, 2) the accurate calibration of flip angles smaller than 20° is important in hyperpolarized VFA MRI (as shown in Fig. 1b).

After acquiring all of the transverse signals from the N excitations, we fit the unknown flip angles using one of two methods. The first method (as shown in Fig. 2c) involves fitting the m angles to Eq. (1) with N = 8, by setting an initial *T*_*1*_ value. In this manner, the m values of θ_1_ ~ θ_m_ are calculated. Then, the residual longitudinal magnetization M_9_ before the 9^th^ excitation can be calculated with the fitted angle value θ_1_ by


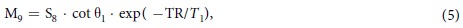


where *T*_*1*_ is the set value, S_8_ is the transverse signal of the 8^th^ excitation with θ_1_. By using of the M_9_ in Eq. (5) and the transverse signal S_9_, a second method (as shown in Fig. 2b) to calibrate flip angles can be gained by


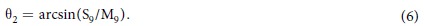


We denote the flip angle θ_2_ calibrated from the first method by Eq. (1) as θ_2_′, and the one calibrated using the second method by Eq. (6) as θ_2_″. If θ_2_′ is equal to θ_2_″, this means that the initial *T*_*1*_ value is correct. If θ_2_′ is not equal to θ_2_″, this means that the initial *T*_*1*_ value is incorrect, and we should change the set *T*_*1*_ to another value. In theory, the *T*_*1*_ can be calibrated from θ_1_ and θ_2_. But due to the measurement error, the *T*_*1*_ calibrated by only θ_1_ and θ_2_ will have significant deviation. Thus if we regard *T*_*1*_ as a constant value during the whole calibration time, the only correct *T*_*1*_ can be derived from the m angles (as shown in Fig. 2d). Once the correct *T*_*1*_ is calibrated, the correct flip angles can be calculated by fitted the data to Eq. (1). Thus, both the flip angles and *T*_*1*_ are obtained from this calculation, and the unknown parameters TG_90°_ and λ in Eq. (3) can also be fitted by the set TGs and calibrated angles. On the other hand, if we neglect the measurement error, we can choose this sequence to determine the change in *T*_*1*_ during breath hold.

### Measurement error simulation

As shown in Figs 1a and 2d, the measured transverse signals are scattered around the fitted value (red line). The mean measurement errors of transverse signals in Figs 1a and 2d are both ±0.7%. In order to test the effect of the measurement error, we use simulations. While in the simulation, the maximum pseudorandom measurement error of transverse signals was adjusted to three values: ±0.5%, ±1.0%, ±1.5%. Each case was repeated 15 times to obtain a dispersion of results. The simulation parameters were set to: TR = 30 ms, *T*_*1*_ = 15 s, TG_90°_ = 10 dB, and λ = 20. The calibration accuracy of the flip angle and *T*_*1*_ was set to 0.02° and 24.5 ms, respectively. The simulated θ_1_ was controlled by TG = 37 dB (θ_1_ = 4.02°), then the transmitter gain was decreased by 1 dB and finally TG_m_ = 24 dB (θ_m_ = 17.96°), with 112 total excitations.

### Materials

**Figure 3:**
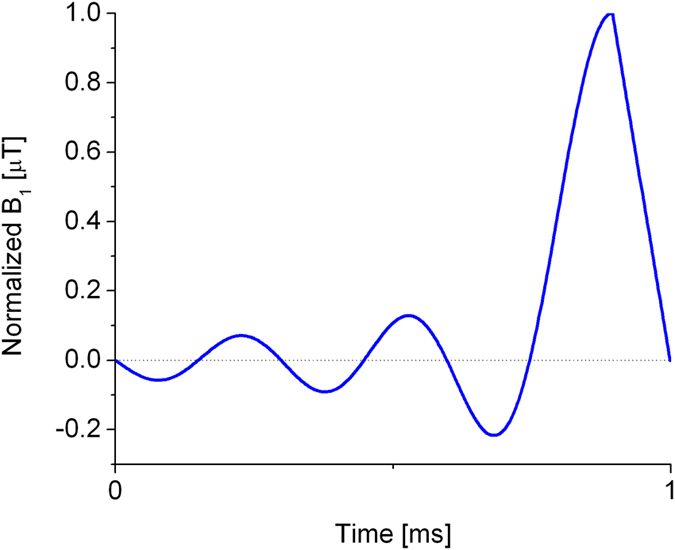
Shape of the RF pulse used in our experiments designed for short TE. The truncated-sinc shape pulse consists of 5 side lobes, a half central lobe and a linear droop.

All experiments were performed on a 7 T animal MRI scanner (Bruker BioSpec 70/20 USR). A homebuilt 8-leg rigid transmit-receive birdcage coil was used. The diameter of the coil was 55 mm and its length was 60 mm. During all experiments, the excitation pulse duration was 1 ms, with the shaped pulse profile shown in Fig. 3. The RF pulse was designed for short TE and good slice selectivity, as slice selective RF pulse could have an issue in VFA experiments with HP samples^33^. The TG_1_ of the pulse in the calibration was set to 30 dB, gradually decreased by 1 dB, and ending at TG_m_ = 16 dB. In each experiment, frequency adjustment and shimming were done during a xenon gas breath hold.

Xenon gas was polarized using a homebuilt continuous-flow polarizer to a level of ~20%. The gas mixture consisted of 2% xenon, 10% N_2_ and balanced ^4^He. In phantom experiments, the xenon gas was natural abundance (~26.4%). For the *in vivo* experiment, the xenon gas was 86% enriched.

After thawing from freezed collection, hyperpolarized xenon gases were collected in a 500 mL Tedlar bag, successively flowed in phantom or lung using a homebuilt ventilator. The breathing sequence contained three parts: 1) Pure oxygen breath with 400 ms inspiration time and 800 ms expiration time. 2) Several xenon gas pre-washes with 500 ms inspiration time and 1000 ms expiration time to wash out the residual oxygen and increase the concentration of xenon gases. 3) Sampling breath, with 500 ms inspiration time, breath-hold time t_hold_ and 1000 ms expiration time. In phantom experiments, t_hold_ was set to 13 seconds. For *in vivo* experiments, t_hold_ was set to 4 seconds. The 500 ms inspiration time can yield a tidal volume of ~2.2 mL. The pressure during breath-hold was 40 cmH_2_O in the balloon and 15 cmH_2_O *in vivo*, due to the larger elasticity of the balloon compared to rat lung. The acquisition was triggered 200 ms after the start of the breath-hold to avoid the influence of gas flow.

Both *T*_*1*_ and flip angles were measured by traditional multi-breath methods to verify the accuracy of the single-breath method. The traditional *T*_*1*_ measurement method consists of varying the delay time between trigger and the 1^st^ RF excitation. with several breaths needed for obtaining different delay times. In traditional flip angle measurement experiments, 16 different flip angles are measured with 16 breaths, and 112 excitations for each angle. The 16 transmitter gains of the RF pulses ranged from 32 dB to 16 dB. The xenon gas pre-wash time was zero in the traditional flip angle measurements, while the pre-wash time was different in *T*_*1*_ measurements to acquire different oxygen concentrations. All traditional measurements had a positive and opposite order, to avoid the signal attenuation from *T*_*1*_ relaxation (~30 min) in the Tedlar bag. The breaths and time needed for a single measurement were shown in Table 1.

The NMR FIDs were transformed to the complex Lorentzian by Fourier transform and phase correction. We chose the integral range from −10 ppm to 10 ppm in the absorption Lorentzian as the actual acquired signal amplitude in all analysis.

### Balloon phantom measurements

In the balloon phantom experiments, the NMR parameters were: TR = 67 ms, sampling points = 1024, reception bandwidth = 18.029 kHz. Therefore, the entire acquisition time was 56.8 ms, which is longer than 5 times the *T*_2_* of ^129^Xe in the phantom. Thus, the residual transverse magnetization at the end of acquisition was quite small, and the truncation effect could be avoided. After the acquisition, a spoiling gradient was applied to the transverse magnetization before the next excitation. During the traditional *T*_*1*_ measurement in phantom experiments, 7 delay times were set to 0 s, 1 s, 2 s, 4 s, 6 s, 9 s, 13 s.

### *In vivo* measurements

Animal experiments were carried out in accordance to guidelines provided and approved by the Institutional Review Board of Wuhan Institute of Physics and Mathematics (WIPM), Chinese Academy of Sciences (CAS). The *in vivo* experiments were performed on Wistar rats. Anesthesia was induced with 5% isoflurane and maintained between 2.5 and 3% isoflurane. Animals were intubated with a 14 G endotracheal tube, tied to the trachea. The NMR parameters were: TR = 33.7 ms, sampling points = 1024, reception bandwidth = 50 kHz. Due to the susceptibility difference between the alveolar gas and the pulmonary tissue, the *T*_2_* of ^129^Xe gas was as short as 3 ms on 7 T. Thus the total acquisition time of 20.48 ms was also longer than 5 times the *T*_2_* of the ^129^Xe gas. After acquisition, a spoiler gradient was applied to the transverse magnetization in preparation for the next acquisition. Due to the small solubility (~1%) of ^129^Xe in tissue/blood, and the submillisecond *T*_2_* of dissolved ^129^Xe, even the reception bandwidth contained the chemical shift of dissolved ^129^Xe, the dissolved ^129^Xe signals were not obtained in all experiments. In order to keep the rats alive, the entire breath hold time cannot be too long. So in the traditional *T*_*1*_ measurement experiments, 6 delay times were set to 0 ms, 250 ms, 750 ms, 1250 ms, 2250 ms, 3750 ms.

## Results

### Simulation results

**Figure 4:**
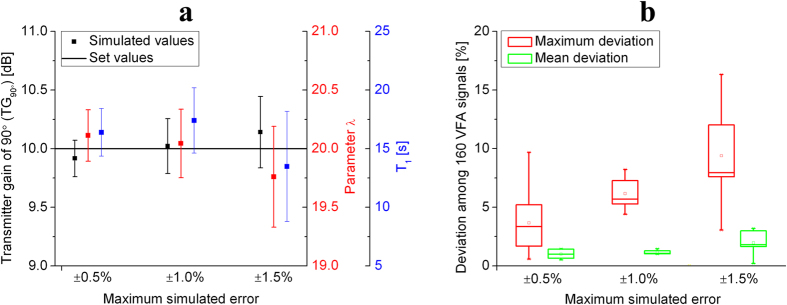
Simulated results of the single-breath method. **(a)** The simulated results of TG_90°_, λ, and *T*_*1*_ are scattered around the set values (10 dB, 20, 15 s, respectively). When maximum simulated error becomes bigger, the repeatability of the single breath method seems to be worse. **(b)** Deviation among 160 VFA signals combined with the simulated calibration results.

The accuracy of simulation is shown in Fig. 4a. The results demonstrate that the single-breath method described above yields repeatable values of both the angle calibration and *T*_*1*_ measurement. The mean values of simulated parameters are very close to the set values. When measurement error increases from ±0.5% to ±1.5%, the deviation of simulated values becomes bigger, which indicated that the repeatability of this method slightly worsened. *In vivo* experiments, the measurement error remains between ±0.5% and ±1.0%, so that the measurement error will have a slight effect on the calibration. To verify the accuracy of TG_90°_, λ, and *T*_*1*_, the parameters were applied to simulate VFA excitations according to Eq. (4). We chose the deviation of transverse signals obtained by 160 VFA excitations as an indicator of the accuracy of simulated parameters. As shown in Fig. 4b, the max deviation of VFA signals may be about 5% for *in vivo* experiments.

### *T*_*1*_ and flip angle measurements

**Figure 5:**
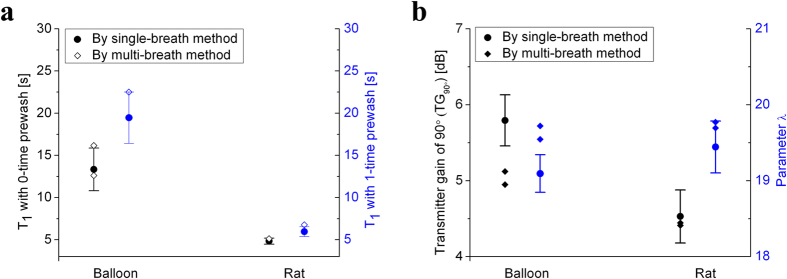
Contrast of measured values by the single-breath method and those by traditional multi-breath method. **(a)** Measured *T*_*1*_ values by single-breath method and traditional multi-breath method. **(b)** Measured transmitter gain of 90° and parameter λ. These results by the single-breath method are close to those by the multi-breath method.

Fig. 5a shows the measured *T*_*1*_ in the balloon phantom and the rat lung, where *T*_*1*_ can be regarded as *T*_*1*_[pO_2_]. In both balloon phantom and rat lung, the measured *T*_*1*_ values with 0-time pre-wash are shorter than that with the 1-time pre-wash. This reflects a larger oxygen pressure pO_2_ in 0-time pre-wash relative to the 1-time pre-wash. After expiration, the residual volume (RV) in balloon is quite small compare to that in the rat lung, due to the bigger elasticity of balloon. Thus the oxygen concentration during the next xenon breath hold was small (~16%) in the balloon, but remained at a high level (~50%) in the rat lung, so that the *T*_*1*_ in rat lung was much shorter than that in the balloon phantom. If we assume that ξ value at 7 T is close to that at 3 T^31^, the pO_2_ can be calculated by Eq. (2). The calculated pO_2_ in balloon and rat lung were close to 200 mbar and 550 mbar, respectively. The *T*_*1*_ value measured by the single-breath method remained very close to that obtained by the traditional method. All single-breath measurements were repeated 5 times. The traditional method was repeated twice for the 0-time pre-wash in balloon, and once for others.

Fig. 5b shows the measured flip angle calibration parameters in Eq. (3), where the xenon pre-wash time was 0 in all measurements. *T*_*1*_ was set to 14 s in the balloon, and 5 s in the rat. The traditional multi-breath measurements were repeated twice, while the single-breath measurements were repeated 5 times in both phantom and rat lung experiments. The parameter λ is close to 20, but not exactly equal to 20. The transmitter gain of 90° in rat lung was smaller than that in the balloon, which is the case because of the RF loss in rat tissues. The measured values from the single-breath method are all close to those obtained by the traditional method, which indicated that the single-breath method was robust.

### VFA excitations

**Figure 6:**
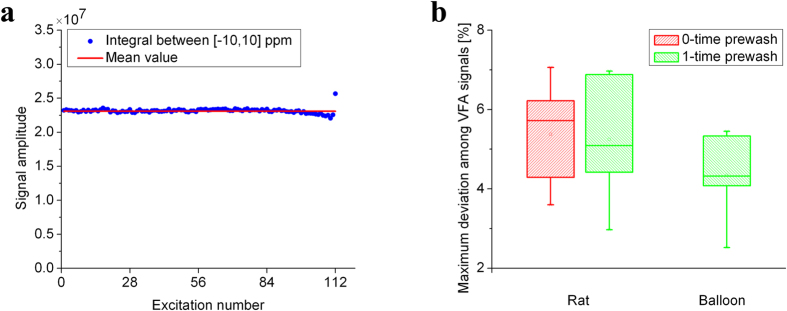
Results of VFA excitations combined with calibration results by single-breath method. **(a)** A typical transverse signals of 112 VFA excitations. All the signals were almost the same except for the final excitation. **(b)** Maximum deviation of VFA signals besides the final excitation in 5 measurements. The maximum deviation of excitations except for the final excitation is scattered around ±5% in all cases.

In order to verify the accuracy of angle calibration and *T*_*1*_ measurement by the single-breath method, the result of calibration has been applied to all VFA excitations. Fig. 6a shows the typical 112 transverse signals in VFA scheme. All the signals were almost the same except for the final excitation. The slight increase of the final excitation may be due to B_1_ inhomogeneity (about 4.6%) of coil, which is similar to others’ VFA work^34^,^35^. Fig. 6b displays the quantitative deviation of VFA signals. The maximum deviation of excitations except for the final excitation is about ±5%, which is consistent with the simulated results from Fig. 4b. Besides, the mean deviation of all excitations is about ±2%, which indicated the repeatability of the calibration results is excellent. All experiments were repeated 5 times, by using the respective results of single-breath calibration.

## Discussion

In this study, a novel method was presented to simultaneously calibrate flip angles and measure *T*_*1*_ of hyperpolarized gas during a short single breath-hold *in vivo*. The method showed good robustness and repeatability. The calibration results of both flip angles and *T*_*1*_ were found to be accurate.

The measured *T*_*1*_[pO_2_] of ^129^Xe gas in rat lung was quite short (5~6 s) in this work, which may be due to the high oxygen concentration from pure oxygen breathing. In human studies, the oxygen concentration in the lung is about 13~19%, similar to the balloon experiment in the present work. However, due to the finite solubility of ^129^Xe in parenchymal tissue, the estimates of pO_2_ via *T*_*1*_ measurements can be ~40% higher than excepted^31^. Thus, we speculate that the measured *T*_*1*_ in human lungs might be 10–15 s. If the repetition time and excitation number was 10 ms and 128, respectively, as the most common cases in hyperpolarized NMR and MRI, the signal decay from RF excitations would be about 8–12%. For patients who suffer from severe lung diseases and need to be given elevated oxygen treatment via ventilator, the oxygen concentration might be higher, resulting in a shorter *T*_*1*_. In this case, the effect of *T*_*1*_[pO_2_] in hyperpolarized MRI can no longer be neglected, and the single-breath calibration method yields to *T*_*1*_ measurements of flip angles within the same breath hold.

Simultaneously with the measurement of *T*_*1*_, the method can also calibrate flip angles. The results of repeatable VFA application indicate the accuracy of those flip angles smaller than 20°. Meanwhile, in further studies, we found that the TG_90°_ calibrated by the single-breath method can gain a flip angle of 90.9° ± 4.4° over 30 repeated measurements. It indicated that this method can also obtain acceptable accuracy for the larger flip angles. The increase of the last excitation in the VFA scheme may be due to the B_1_ inhomogeneity of coil, just like other’s VFA works^34^,^35^. In this study, we have not applied any gradient to achieve a slice selection. Therefore, the unusually large signal of the final excitation in our VFA applications is not due to the issues of slice selective pulse. The measured parameter λ in flip angle calibration was scattered around 20. Considering the same phenomenon in measurement error simulation, we speculate that the random values of λ may be caused by the measurement error or fitting error. In any case, the method we proposed in this work can correct this error and gain accurate VFA signals.

Despite the advantages of this single-breath method, there remain limitations to this work. First, the 4 s acquisition times are still too long. An improved solution by decreasing the sampled points from 1024 to 256 may work. This would decrease the acquisition time fourfold. Second, we regard the flip angle and *T*_*1*_ in 4 s acquiring time as a constant value. During *in vivo* experiments, *T*_*1*_ is not constant^36^ due to the perfusion and consumption of O_2_. In reality, the flip angle is also inhomogeneous across the whole lung due to the B_1_ field inhomogeneity. The variation of *T*_*1*_ within the 4 s period was difficult to correct as *T*_*1*_ is spatially dependent. If the measurement error and acquisition time were small enough, we may have the possibility to measure *T*_*1*_ from only θ_1_ and θ_2_, making it possible to measure the variation of *T*_*1*_ during breath-hold. However, that is unlikely to be possible. For short acquisition times, we can also measure the flip angles and *T*_*1*_ distribution in different slices by adding slice selected gradients. Thirdly, we only tested the hyperpolarized ^129^Xe method in small animals. This same problem also affects other hyperpolarized media like ^13^C, ^3^He. The verification will be left for further studies. During human experiments, the acquisition time should be shorter due to the variation of *T*_*1*_ during breath-hold.

## Conclusion

It was shown that the single-breath method proposed in this work is an effective tool to simultaneously calibrate flip angles and measure *T*_*1*_ of hyperpolarized gas during a short single breath-hold *in vivo*. The method shows good robustness and repeatability, especially important for cases of short *T*_*1*_. Different from the published flip angle calibration methods for hyperpolarized media, this method does not need to neglect or avoid the influence of *T*_*1*_. The flip angle calibration by this method is accurate for the angles smaller than 20°, and the inaccuracy for large angles is also acceptable. The method is suitable for different type of coils and also acceptable for low SNR (big measurement error) experiments. Therefore, the method can provide accurate and rapid flip angle calibration and simultaneously *T*_*1*_ measurement for different volunteers, patients or small animals. And the method can also yield stable transverse signals for more important applications like UTE, CVFA^37^ sequences. The calibration time needed for this method was less than 4 s *in vivo* and can be further shortened. We obtained repeatable steady state transverse signals by using this single-breath method, and the mean deviation of transverse signals was found to be ~2%.

## Additional Information

**How to cite this article**: Zhong, J. *et al.* Fast Determination of Flip Angle and T_1_ in Hyperpolarized Gas MRI During a Single Breath-Hold. *Sci. Rep.* **6**, 25854; doi: 10.1038/srep25854 (2016).

## Figures and Tables

**Table 1:** Breaths and time needed *in vivo* for a single measurement by the two methods used in this work.

	Traditional multi-breath method	Single-breath method
Breaths	6 for *T*_1_ measurement, 16 for FA calibration	1 for all
Time	88 s	3.8 s
